# Monitoring Prevalence and Persistence of Environmental Contamination by SARS-CoV-2 RNA in a Makeshift Hospital for Asymptomatic and Very Mild COVID-19 Patients

**DOI:** 10.3389/ijph.2023.1605994

**Published:** 2023-09-12

**Authors:** Jinyan Yang, Dan Sun, Tingting Xia, Shi Shi, Jijiang Suo, Huihui Kuang, Nana Sun, Hongyan Hu, Zhecheng Zheng, Yang Zhou, Xiaocui Li, Shaojuan Chen, Haiqiang Huang, Zhongqiang Yan

**Affiliations:** ^1^ Department of Disease Prevention and Control, Hainan Hospital of People’s Liberation Army of China General Hospital, Sanya, China; ^2^ Department of Laboratory Medicine, Hainan Hospital of People’s Liberation Army of China General Hospital, Sanya, China; ^3^ Department of Health Economics Management, Hainan Hospital of People’s Liberation Army of China General Hospital, Sanya, China; ^4^ Department of Cardiology, Hainan Hospital of People’s Liberation Army of China General Hospital, Sanya, China; ^5^ Department of Radiotherapy, Hainan Hospital of People’s Liberation Army of China General Hospital, Sanya, China; ^6^ Department of Disease Prevention and Control, The Second Medical Center of People’s Liberation Army of China General Hospital, Beijing, China

**Keywords:** COVID-19, SARS-CoV-2, makeshift hospital, environmental contamination, RT-qPCR

## Abstract

**Objective:** To investigate the details of environmental contamination status by SARS-CoV-2 in a makeshift COVID-19 hospital.

**Methods:** Environmental samples were collected from a makeshift hospital. The extent of contamination was assessed by quantitative reverse transcription polymerase chain reaction (RT-qPCR) for SARS-CoV-2 RNA from various samples.

**Results:** There was a wide range of total collected samples contaminated with SARS-CoV-2 RNA, ranging from 8.47% to 100%. Results revealed that 70.00% of sewage from the bathroom and 48.19% of air samples were positive. The highest rate of contamination was found from the no-touch surfaces (73.07%) and the lowest from frequently touched surfaces (33.40%). The most contaminated objects were the top surfaces of patient cubic partitions (100%). The median Ct values among strongly positive samples were 33.38 (IQR, 31.69–35.07) and 33.24 (IQR, 31.33–34.34) for ORF1ab and N genes, respectively. SARS-CoV-2 relic RNA can be detected on indoor surfaces for up to 20 days.

**Conclusion:** The findings show a higher prevalence and persistence in detecting the presence of SARS-CoV-2 in the makeshift COVID-19 hospital setting. The contamination mode of droplet deposition may be more common than contaminated touches.

## Introduction

In early August 2022, Sanya had been reporting a high number of COVID-19 cases caused by the Omicron subvariant BA.5.1.3 and had registered over 4,232 laboratory-confirmed COVID-19 infectors as of 11 August 2022. At the crucial stage, construction workers from across Hainan Province raced against time to construct a makeshift hospital. The second makeshift COVID-19 hospital, converted from the international expo center project (Sanya, China), covered approximately 21,200 square meters with a capacity of 2,000 beds and was delivered on 11 August 2022. In the makeshift hospital, COVID-19 patients with asymptomatic or mild symptoms were placed in the patient care areas (quarantine center) [1, 2], with natural ventilation by open windows and ceiling fans and an air exchange rate of less than 5 times per hour. Makeshift hospitals can effectively respond to the COVID-19 epidemic by performing essential functions such as triage [3], isolation, sheltering, and rapid transfer [2]. In the meantime, they have some limitations, e.g., difficulties with heating, ventilation, and air conditioning systems [4]. Sufficient and effective ventilation can be possibly enhanced by particle filtration and air disinfection, avoiding air re-circulation, and avoiding overcrowding. A makeshift hospital could yield a higher success rate for surface swab samples due to a substantially less stringent cleaning and disinfection regime.

According to current evidence, SARS-CoV-2 has a high infector-to-environment contamination rate through close contact with infected individuals, especially when the viral shedding of SARS-CoV-2 occurs during breathing, talking, coughing, or sneezing from symptomatic, asymptomatic, and presymptomatic persons [5]. Many surveys have investigated the presence of SARS-CoV-2 RNA contamination in a wide range of facilities, settings, and wastewater [6–8]. Persistent contamination of SARS-CoV-2 RNA may be related to the “3Cs” that the expert panel for COVID-19 in Japan focused on, namely, “Closed spaces with poor ventilation,” “Crowded spaces with many people,” and “Close contact” [9]. In addition, SARS-CoV-2 can persist on environmental surfaces for extended periods, sometimes up to months. Taken together, recent aggregated studies suggest that SARS-CoV-2 RNA can be readily detected on surfaces and fomites [10], and strengthen the possibility of contaminated environmental samples for SARS-CoV-2 transmission [11]. Notably, the virus SARS-CoV-2, the causative agent of COVID-19, can be acquired by exposure to fomites, although the infectious risk through fomites is probably multifactorial and is affected by several environmental stressors, including humidity, temperature, and ventilation [12].

Several studies have shown that different extents of SARS-CoV-2 contamination vary from no contamination to low or high contamination by viral RNA. Additionally, most of the reported positive environmental samples were found to have high RT-qPCR cycle threshold (Ct) values (>30) for most of the positive samples [13], indicating a low viral load and the liable nature of SARS-CoV-2 in the environment. Laboratory-confirmed COVID-19 infectors can release particles and droplets of respiratory fluids that contain the SARS-CoV-2 virus into the air when they exhale [14]. The principal mode by which environments are contaminated with SARS-CoV-2 is through exposure to respiratory fluids carrying infectious virus [15]. The extent and mode of environmental contamination with SARS-CoV-2 RNA in a makeshift hospital are poorly understood. This study experimentally characterized SARS-CoV-2 on several types of environmental surfaces, wastewater from bathrooms, and air samples deployed in patient care areas, and aimed to investigate the prevalence, persistence, and load of viral RNA in a makeshift COVID-19 hospital which was located in Sanya (China) with a tropical climate.

## Methods

### Makeshift Hospital Settings

The second Sanya makeshift hospital, converted from the international expo center project, was divided into different sections to avoid COVID-19 cross-infection. The section for quarantining infected patients was divided into four pods, A, B, C, and D, with a capacity of 336, 608, 606, and 336 bed units, respectively. Each pod was divided into several units composed of 30–40 beds. A total of 320 healthcare workers (HCWs) in rotation entered the pods for a shift of 4 h. There were 60–80 camera detectors installed on the top of patient cubic partitions in each pod. The makeshift hospital was initiated to use on August 11, 2022, and operated for nearly 60 days, admitting 7,192 patients who predominantly had mild COVID-19 or asymptomatic cases. “Zero infection” of HCWs was achieved. With the stabilization of the epidemic and the decrease in bed occupancy, the remaining COVID-19 infectors were discharged by 10 October 2022, and no deaths were reported.

### Sampling Strategy

This prospective study was conducted from 16 August to 6 October 2022 at the second Sanya makeshift COVID-19 hospital, in China. This work experimentally characterized SARS-CoV-2 on several types of environmental surfaces, wastewater from bathrooms, and air samples deployed in patient care areas (Table 1). All surface sampling was performed in the morning before the first cleaning cycle for the day and the frequency of sample collection was once a day. A consistent sampling method was used by the same study investigators across different pods and patient bed units for each sampling location. All samples were taken to the laboratory of the makeshift hospital within 1 h post-sampling and processed on the day of collection.

**TABLE 1:** Pre-assigned sampling points in patient care areas for detection of SARS-CoV-2 RNA (Sanya, China, August 2022).

Types of samples	Sampling sites
Frequently touched surfaces	1. Trash bin top, 2. All surfaces of tables and chairs in staff workstations, 3. Floor surface, 4. Door handle, 5. Staff computer mouse and keyboard, 6. Gloves in contact with patient surrounding, 7. Medical equipment, 8. Bedrail, 9. Bedside table, 10. Patient bedding (pillow, bedsheet, and duvet), 11. Patient daily necessities, 12. Patient mobile phone, 13. Inner surface of the patient’s mask, 14. Patient’s hand
No-touch surfaces	15. The top surface of patient cubic partitions, 16. The top surface of the power cable storage box, 17. Camera detector top, 18. Floor beneath the patient bed, 19. Top of fire extinguisher surface, 20. Windowsill, 21. Grills of portable air cleaner
Toilet setting	22. Faucet handle, 23. Toilet countertops, 24. Sewage from bathroom
Air	25. Active air sampling (one centre and four corners), 26. Deposition air sampling (the top of the partition wall of the patient bed space)

### Environmental Surface Sampling Procedure

Surface sampling was conducted using flocked sterile swabs (MS-OF3601, Shenzhen MandeLab Co., Ltd.) pre-moistened with viral transport medium (VTM). The tip of the swab was dipped in a PP tube containing VTM (CIDA, Guangzhou, Biotechnology Co., Ltd.) and then was rubbed by moving the swab in two different directions while rotating the stick with gentle pressure over a recommended surface area of the study surfaces. After swabbing the surface, the swab tip was preserved in a pre-labeled 10 mL disposable virus-inactivated specimen collection tube (CIDA, Guangzhou, Biotechnology Co., Ltd.).

Frequently touched and no-touch surface samples were taken from pre-assigned locations (Table 1). The areas of the sample surfaces, which were made of plastic, painted wood, metal, or cement floor, ranged from approximately 30–100 cm^2^. The area of the flat surfaces sampled was kept constant at 100 cm^2^ by sliding and rotating a moistened swab. For the non-flat surfaces, any available area was sampled for different sampling sites, due to the nature and shape of the objects.

### Air Sampling Procedure

To determine the presence of SARS-CoV-2 particles in the air, following the study protocol, the indoor air of the makeshift hospital was sampled simultaneously by two different methods, air sampler and natural sedimentation. Active air sampling was conducted with filter-based TH-150H air samplers (Wuhan Tianhong Instruments, Wuhan, China). Air samplers were placed on tripods at 1.5 m above the ground and at least 2 m away from the patient cubic partitions. Five air samplers were deployed in the center and four corner points within the patient care area of each pod in each air sampling campaign. Total suspension particulate (TSP) matter samples were collected on 90 mm quartz filters (Pallflex Tissuquartz, Pall, Port Washington, NY, USA) over a period of 4 h using a TH-150H medium volume air sampler at a flow rate of 100L/min, each air sample represents 24,000 L of air. At the end of air sampling, each whole quartz filter was soaked into 5 mL VTM and incubated at 37°C for 10 min. After the dissolution of the quartz filter, 1 mL VTM was collected for nucleic acid extraction. Airborne settling dust was sampled using 10 mL PP tubes containing 3 mL VTM (CIDA, Guangzhou, Biotechnology Co., Ltd.), which were open and exposed to air for 2 days to collect biological particles that deposited themselves in the tubes. PP tubes were placed in holders pinned to the top of the partition walls of the patient bed space, approximately 2 m above the floor level.

### Sewage Sampling Procedure

The makeshift hospital was a temporary building with a poor drainage system; there was a lot of sewage around the sewer catchment. We used dry-flocked swabs to collect wastewater from the sewer catchment in the patient’s bathroom. The tip of the swab was immersed in the sewage for 2 min, and then the moistened swab was inserted into the PP tube containing 3 mL VTM.

### Longitudinal No-Touch Surfaces Monitoring for SARS-CoV-2 Persistence Assay

After pod D was vacated (on 15 September 2022), settling dust swabbing samples were collected once every 4 days from 19 September to 5 October 2022 (Supplementary Table S2). The swabbed surfaces were out of reach and non-cleaned, including windowsills, bedrails, bedside tables, camera detector tops, top of fire extinguisher surfaces, and the floors beneath the patients’ beds, and a different part of these surfaces was swabbed each time. Most swabs took a very dark color from the dust they collected.

### Laboratory Procedures for Detection of SARS-CoV-2 RNA

Environmental samples were vortexed vigorously for 20 s before aliquoting. Ribonucleic acid extractions were performed using a DaAn Gene nucleic acid extraction kit (DaAn Gene Co, Ltd., of Sun Yat-sen University, China) according to the manufacturer’s instructions. RT-qPCR was performed using a DaAn Gene 2019-nCoV kit (DaAn Gene Co, Ltd., of Sun Yat-sen University, China). Two separate gene targets, the open reading frame 1a/1b (ORF1ab) and the nucleocapsid protein (N) genes, were used to detect SARS-CoV-2 RNA. The PCR tubes were immediately transferred to an ABI 7500 RT-qPCR machine (Applied Biosystems Inc., Foster, CA, United States). The cycling conditions were as per the manufacturer’s protocol. A sample was defined as strongly positive for viral RNA if both the ORF1ab and N RT-qPCR assays gave Ct ≤40, and negative when they were both >40. If only one of the target genes had a Ct value ≤40 and the other >40, it was interpreted as a single-gene positive (weakly positive).

### Statistical Analysis

**FIGURE 1:**
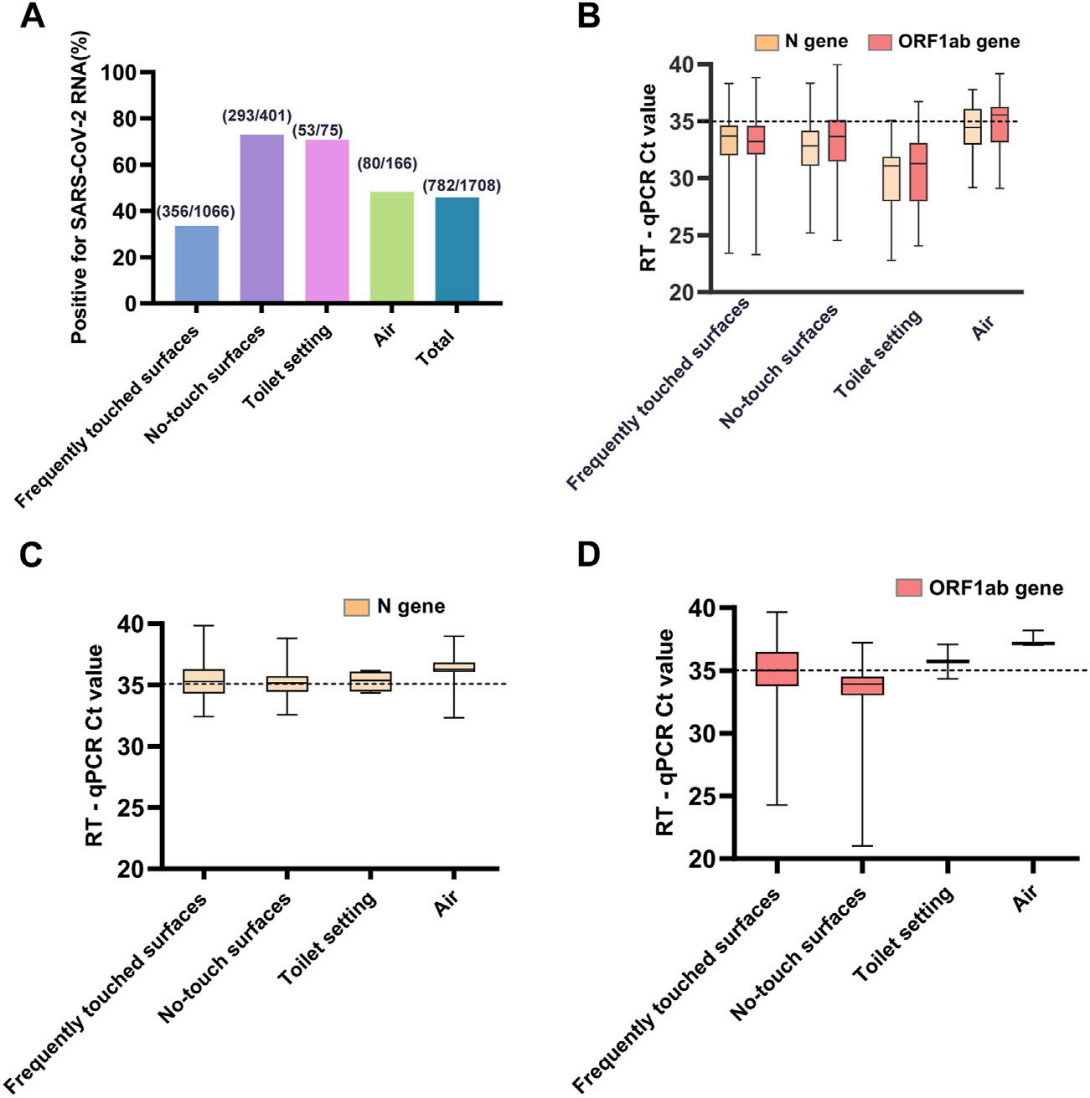
**(A)** The proportions of RT-qPCR-positive samples for each type samples and total samples of the four types. **(B)** The trajectory of Ct values of ORF1ab and N genes for strongly positive environmental samples. **(C)** The trajectory of only Ct values of N gene for weakly positive environmental samples. **(D)** The trajectory of only Ct values of ORF1ab gene for weakly positive environmental samples. A sample was defined as strongly positive for viral RNA if both ORF1ab and N RT-qPCR assays gave Ct ≤ 40, if either ORF1ab or N gene had a Ct value ≤40 and the other >40, it was interpreted as a weakly positive (Sanya, China, August 2022).

The results from PCR were recorded onto Excel Spreadsheet. Statistical analysis was performed using IBM SPSS 23.0 software. Continuous variables were presented as the median and interquartile range (IQR), and comparisons between groups were analyzed with Student’s t-test (normal distribution) or nonparametric Kruskal-Wallis H rank-sum (skewed distribution). Categorical variables were presented as counts and percentages, and differences between groups were analyzed with χ2 or Fisher’s exact test. *p* < 0.05 (two-sided) was considered statistically significant. The distribution of Ct values for RT-qPCR-positive samples is represented by a box chart (Figures 1B, C, D).

## Results

### Prevalence of Environmental Samples Contamination With SARS-CoV-2 RNA

During the collection period, we performed 2,167 sample collections, comprising 1,708 samples for investigating the prevalence of SARS-CoV-2 RNA in the makeshift COVID-19 hospital setting, and 435 samples for monitoring the persistence. Our results showed a high success rate in detecting the presence of SARS-CoV-2 RNA in the makeshift COVID-19 hospital setting. All four types of collected samples tested positive (Table 2). There was a wide range in terms of the total collected samples contaminated with SARS-CoV-2 RNA, ranging from 8.47% to 100%. The highest rate of contamination with SARS-CoV-2 RNA was found from the no-touch surfaces (73.07%) and toilet setting (70.67%), followed by the air samples (48.19%), and the lowest rate was from frequently touched surfaces (33.40%) (Figure 1A). There was a statistically significant difference in the proportions of RT-qPCR-positive samples between the four sample types (χ^2^ = 205.26, *p* < 0.001).

**TABLE 2:** Environmental sampling results (Sanya, China, August 2022).

Type of sample	Number of samples collected (%)	Number of positive (%)	Strongly positive samples (%)	Weakly positive samples (%)	χ^2^ or Fisher’s exact test	*p*
Frequently touched surfaces	1,066 (62.41)	356 (33.40)	247 (69.38)	109 (30.62)	χ^2^ = 251.923	<0.001
1. Trash bin top	33 (3.10)	26 (78.79)	23 (88.46)	3 (11.54)		
2. All surfaces of tables and chairs in staff workstations	58 (5.44)	43 (74.14)	25 (58.14)	18 (41.86)		
3. Floor surface	45 (4.22)	33 (73.33)	20 (60.61)	13 (39.39)		
4. Door handle	34 (3.19)	21 (61.76)	17 (80.95)	4 (19.05)		
5. Staff computer mouse and keyboard	11 (1.03)	5 (45.45)	5 (100.00)	0 (0.00)		
6. Staff gloves in contact with patient surrounding	45 (4.22)	22 (48.89)	17 (77.27)	5 (22.73)		
7. Medical equipment surface	54 (5.07)	41 (75.93)	30 (73.17)	11 (26.83)		
8. Bedrail	52 (4.88)	13 (25.00)	10 (76.92)	3 (23.08)		
9. Bedside table	77 (7.22)	26 (33.77)	18 (69.23)	8 (30.77)		
10. Patient bedding (pillow, bedsheet, and duvet)	97 (9.10)	36 (37.11)	26 (72.22)	10 (27.78)		
11. Patient daily necessities	206 (19.32)	45 (21.84)	26 (57.78)	19 (42.22)		
12. Patient mobile phone	82 (7.69)	8 (9.76)	7 (87.50)	1 (12.50)		
13. Inner surface of the patient’s mask	213 (19.98)	32 (15.02)	21 (65.63)	11 (34.38)		
14. Patient’s hand	59 (5.53)	5 (8.47)	2 (40.00)	3 (60.00)		
No-touch surfaces	401 (23.48)	293 (73.07)	220 (75.09)	73 (24.91)	χ^2^ = 46.551	<0.001
15. Top surface of patient cubic partition	14 (3.49)	14 (100.00)	12 (85.71)	2 (14.29)		
16. Top surface of power cable storage box	10 (2.49)	7 (70.00)	6 (85.71)	1 (14.29)		
17. Camera detector top	77 (19.20)	71 (92.21)	61 (85.92)	10 (14.08)		
18. Floor beneath the patient bed	100 (24.94)	78 (78.00)	61 (78.20)	17 (21.79)		
19. Top of fire extinguisher surface	9 (2.24)	5 (55.56)	2 (40.00)	3 (60.00)		
20. Windowsill	149 (37.16)	84 (56.38)	51 (60.71)	33 (39.29)		
21. Grills of portable air cleaner	42 (10.47)	34 (80.95)	27 (79.41)	7 (20.59)		
Toilet setting	75 (4.39)	53 (70.67)	47 (88.68)	6 (11.32)	χ^2^ = 0.981	0.655
22. Faucet handle	8 (10.67)	5 (62.50)	5 (100.00)	0 (0.00)		
23. Toilet countertops	7 (9.33)	6 (85.71)	6 (100.00)	0 (0.00)		
24. Sewage from bathroom	60 (80.00)	42 (70.00)	36 (85.71)	6 (14.29)		
Air	166 (9.72)	80 (48.19)	50 (62.50)	30 (37.50)	χ^2^ = 20.733	<0.001
25. Active sampling	60 (36.00)	43 (71.67)	33 (76.74)	10 (23.26)		
26. Deposition sampling	106 (64.00)	37 (34.91)	17 (45.95)	20 (54.05)		
Total	1708 (100)	782 (45.78)	564 (72.12)	218 (27.88)	χ^2^ = 205.255	<0.001

SARS-CoV-2 was detected in 356 (33.40%) of 1,066 frequently touched surfaces, most frequently on trash bin tops (26/33; 78.79%), medical equipment surfaces (41/54; 75.93%), all the surfaces of the tables and chairs in staff workstations (43/58; 74.14%), and floor surfaces (33/45; 73.33%). The rate of positivity was higher for no-touch surfaces (73.07%) than those frequently touched (33.40%) by HCWs or patients (χ^2^ = 185.91, *p* < 0.001). Among the no-touch surfaces, SARS-CoV-2 RNA was most frequently detected from the top surface of the patient cubic partitions (14/14; 100%), camera detector tops (71/77; 92.21%), grills of portable air cleaners (34/42; 80.95%), and floors beneath the patients’ beds (78/100; 78.00%).

Three types of samples were collected from toilet settings in the patient care areas. The prevalence of SARS-CoV-2 RNA was 62.5% (5/8), 85.71% (6/7), and 70.00% (42/60) for faucets, toilet countertops, and sewage from sewer catchment in the patient bathroom, respectively. There was no statistically significant difference in the proportions of RT-qPCR-positive samples (χ^2^ = 0.98, *p* = 0.655, Fisher’s exact test).

Altogether, 80/166 (48.19%) positive air samples were collected with two methods, including 43/60 (71.67%) from air samplers and 37/106 (34.91%) from natural sedimentation. There was a statistically significant difference between the two different air sampling strategies (χ^2^ = 20.73, *p* < 0.001).

### Distribution of Ct Values for RT-qPCR-Positive Samples

Among the RT-qPCR positive samples, 564/782 (72.12%) environmental samples collected were strongly positive for both ORF1ab and N genes of SARS-CoV-2 RNA, 218/782 (27.88%) were weakly positive for either ORF1ab or N gene. The median Ct values among the strongly positive samples (Figure 1B) were 33.38 (IQR, 31.69–35.07) and 33.24 (IQR, 31.33–34.34) for the ORF1ab and N genes, respectively (Supplementary Table S1). There was a statistically significant difference in the Ct values of the ORF1 (H = 48.781, *p* < 0.001) and N (H = 64.889, *p* < 0.001) genes between the strongly positive samples using the Kruskal-Wallis H test. The median Ct values among the weakly positive samples (Figures 1C, D) were 34.66 (IQR, 33.65–36.90) and 35.36 (IQR, 34.62–36.29) for ORF1ab and N genes, respectively. The detection rate of the N gene (738/1708, 43.21%) was higher than the ORF1ab gene (608/1708, 35.60%), and there was a statistically significant difference (χ^2^ = 20.72, *p* < 0.001).

### Persistence of SARS-CoV-2 RNA in a Vacated Pod of Makeshift COVID-19 Hospital

SARS-CoV-2 relic RNA could be detected on indoor surfaces for up to 20 days after the COVID-19 infectors left the makeshift hospital. A total of 371/435 (85.29%) positive swabbed samples from surfaces that were out of reach and non-cleaned were collected, including 89/114 (78.07%) from windowsills, 45/48 (93.75%) bedrails, 49/66 (74.24%) bedside tables, 75/77 (97.40%) camera detector tops, 41/46 (89.13%) top of fire extinguisher surfaces, and 72/84 (85.71%) floors beneath the patients’ beds.

**FIGURE 2:**
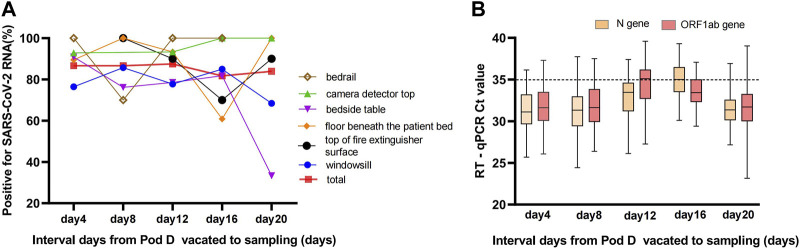
**(A)** Positivity rate of swabbed samples from surfaces that were out of reach and non-cleaned over time up to 20 days. The total curve indicates the detection rate of SARS-CoV-2 RNA for swabbed environmental samples pool at different time points with an steady contamination rate, and the environmental samples pool comprised windowsill, bedrail, bedside table, camera detector top, top of fire extinguisher surface, and floor beneath the patient bed. **(B)** The trajectory of Ct values of ORF1ab and N genes for positive environmental samples during 20 consecutive days (Sanya, China, August 2022).

The frequency of positive samples per sampling episode changed a little over time up to 20 days after pod D was closed. There was no statistically significant difference between the four intervals, each 4 days long (χ^2^ = 1.683, *p* = 0.797, Fisher’s exact test), although the detection rate of positive samples from windowsills and bedside tables slowly decreased on the 20th day (Figure 2). In total, 80.59% (299/371) of the positive samples for SARS-CoV-2 RNA by the RT-qPCR assays were strongly positive for both the ORF1ab and N genes. The median Ct values of the strongly positive samples were 33.39 (31.22–35.33) and 32.46 (30.67–34.11) for the ORF1ab and N genes, respectively (Supplementary Table S2). The median Ct values among the weakly positive samples were 34.70 (IQR, 33.78–36.39) and 35.56 (IQR, 34.86–36.28) for the ORF1ab and N genes, respectively (Figure 2). The detection rate of the N gene (361/435, 82.99%) was higher than the ORF1ab gene (308/435, 70.80%), and there was a statistically significant difference (χ^2^ = 18.17, *p* < 0.001).

## Discussion

A potential role of the present study is to guide the prevention and control of SARS-CoV-2 environmental contamination. Controlling contamination is necessary to reduce virus transmission. Studying the mechanism of environmental contamination in the patient care areas could help to better understand the routes of transmission of SARS-CoV-2. Now it is not the time to relax, we are equipped and prepared to fight the Omicron variant head-on. We are seeing this as a variant of concern and the way to address variants is to slow SARS-CoV-2 transmission, and we do that through makeshift COVID-19 hospitals, and through masking and continuing to apply tried-and-tested public health measures. The contamination of SARS-CoV-2 is likely to occur through a number of modes. Besides direct touching, coughing, sneezing, and speaking could cause environmental contamination by generating droplets or aerosolizing the respiratory fluid [16, 17]. Armed with 3 year’s worth of data about environmental contamination with SARS-CoV-2 in hospital settings, it is clear that airborne transmission should be the main cause for concern in a crowded indoor space [18, 19]. No-touch surfaces and toilets (bathrooms) were important areas in SARS-CoV-2 contamination control, especially in special environmental conditions with low air exchange rates and constant air re-circulation. Environmental surfaces pose an infection risk of contamination with a high viral load of SARS-CoV-2, especially in indoor environments with insufficient ventilation and inadequate filtration. It is important to highlight here that SARS-CoV-2 has many potential environmental contamination pathways, some of them have been established, and many others are yet to be confirmed [20–22].

Recent studies and experimental results on the environmental prevalence and persistence of SARS-CoV-2 have indicated the possibility of environmental transmission via fomites and the air in the vicinity of infected persons and this further reinforced the importance of monitoring environmental contamination [23, 24]. In this study, we analyzed SARS-CoV-2 viral RNA on various surfaces, and in indoor air and bathroom sewage collected simultaneously from COVID-19 patient care settings in a makeshift hospital using RT-qPCR. The high positivity rate, ranging from 8.47% to 100%, suggests that the overall potential risk of environment-based transmission in the tested makeshift COVID-19 hospital was very high. The median Ct values among the strongly positive samples were 33.38 (IQR, 31.69–35.07) and 33.24 (IQR, 31.33–34.34) for the ORF1ab and N genes, respectively. In the present study, the Ct values of all the samples were >30, indicating that the overall risk of infection was not high. Interestingly, the sampled locations with higher contamination rates also had lower Ct values, indicating that the highly contaminated environmental sites may have higher SARS-CoV-2 viral loads.

The lowest rate of contamination with SARS-CoV-2 RNA was from frequently touched surfaces (33.40%) compared to no-touch surfaces (73.07%), toilet setting (70.67%), and air samples (48.19%). In the present study, insufficient and ineffective ventilation was associated with a decreased and increased SARS-CoV-2 contamination rate of frequently touched surfaces and no-touch surfaces, respectively. In the makeshift COVID-19 hospital, frequently touched surfaces were cleaned and disinfected daily, and good hygienic practices appeared to reduce the prevalence of high- or low-touch surface contamination. Several other studies reported that 50%–70% of the masks collected from patients had RT-qPCR-positive results [25, 26]. Unexpectedly, the positivity of the inner surfaces of patients’ masks was only 15.02% (32/213) in the present study. These inconsistent findings may be related to the patient profiles, duration of mask-wearing, sampling methods, and the sensitivity of the technique used. We agree that the presence of SARS-CoV-2 RNA on frequently touched surfaces could be through the deposition of droplets, in addition to contaminated touches [27]. The greatest frequency of viral contamination was actually on the no-touch surfaces (73.07%), suggesting that the contamination mode of droplet deposition was more common than contaminated touches in the second Sanya makeshift COVID-19 hospital. We can conclude that the principal mode by which environmental materials were contaminated with SARS-CoV-2 was through exposure to respiratory aerosols of different sizes carrying the infectious virus. Many frequently touched surfaces were in close proximity to the COVID-19 infectors and could be contaminated by a wide range of droplets from patients during breathing, speaking, coughing, or sneezing, and not necessarily contaminated by touch. Moreover, this work also demonstrated that floor or windowsill dust was a potentially useful matrix for long-term surveillance of respiratory particles containing the virus in designated settings for treating high-risk populations.

The present study has shown that SARS-CoV-2 RNA was detected in 70.00% (42/60) of the sewage samples from the sewer catchment in the patient bathroom of the makeshift hospital of COVID-19. Monitoring the SARS-CoV-2 RNA in samples of wastewater from the patient’s bathroom remains important to avoid healthcare workers’ occupational exposure to the bioaerosols from sewage produced by COVID-19 infectors [28, 29]. We successfully implemented the use of flocked swabs in the detection of SAR-CoV-2 RNA in wastewater. Flocked swab sampling, although simple and convenient, provides a snapshot of the representation of the wastewater system in the makeshift COVID-19 hospital [30]. The risk of wastewater transmission can be reduced by following strategies: (1) improving the design and maintenance of sewers in makeshift hospitals and avoiding spilling wastewater into the environment; (2) using chemical and other disinfection processes to remove the virus before discharging into the sewage line; (3) wastewater surveillance for SARS-CoV-2 can be performed by collecting sewage samples at the point of initial discharge into the local sewage system; and (4) increasing the wastewater sample collection frequency from weekly to daily, and monitoring the viral load for SARS-CoV-2 RNA in the wastewater for the prior detection of a COVID-19 outbreak can guide timely targeted interventions.

Positive results for SARS-CoV-2 RNA in the air have been found with all the different known methods available, such as filter-based samplers, impingers, impactors, cyclones, water-based condensation, and passive sampling [31–34]. In the present study, indoor air SARS-CoV-2 particles of the makeshift hospital were sampled simultaneously by two different methods, air sampler and natural sedimentation [35–37]. The air sampling results of this study showed that 48.19% (80/166) of air samples were positive for SARS-CoV-2 by RT-qPCR, moreover, air samplers (43/60, 71.67%) were more efficient than natural sedimentation (37/106, 34.91%). These results suggest that the two sampling methods used are suitable for detecting SARS-CoV-2 RNA in air samples. A large number of positive air samples improved our ability to identify risk factors for the presence of SARS-CoV-2 in aerosol samples and furthered our understanding of the dispersion dynamics of SARS-CoV-2 in patient care areas with implications for the occupational exposure of healthcare workers.

This study also aimed to determine the persistence of SARS-CoV-2 on the non-cleaned surfaces (fomites) in the vacated pod D. The results showed that SARS-CoV-2 RNA can persist for up to 20 days (Figure 2A) on several indoor surface materials and a high positivity rate (83.95%) remained. It has been reported that SARS-CoV-2 can remain in the environment for a longer time [38, 39], but not necessarily as an infectious virus, possibly as a “relic RNA” virus. This study further suggested that it was difficult to distinguish whether any detectable RNA in the environment was from recent deposition or represents relic RNA. One challenge with environmental monitoring of SARS-CoV-2 is how to account for the intricate relationship between contamination and transmission of SARS-CoV-2 through novel approaches and perspectives.

It is suspected that contaminated surfaces or fomites also have a role in transmission [40–43]. Fomites, inanimate objects capable of absorbing, harboring, and transmitting infectious agents, have been implicated as possible sources of transmission [44–46]. According to biosafety regulations and the limited resources of the makeshift hospital, virus culturing was not performed to isolate live SARS-CoV-2 from any of our collected samples in this study. Of course, it is important to determine the potential risk of fomites transmission, and future studies should focus on isolating the virus from various surfaces, indoor air, and bathroom sewage through funding and design support for COVID-19 projects. Some studies have shown a lack of positive viral cultures and suggested that the risk of transmission of SARS-CoV-2 through fomites is low [47, 48]. Heterogeneity in study designs and methodology prevents comparisons of findings across studies. Standardized guidelines for conducting and reporting research on formite transmission are warranted. Overall, our findings showed a higher contamination rate in environmental settings, suggesting comprehensive infection control measures should be taken to minimize the risk of infection among the exposed HCWs. Ensuring adequate ventilation by increasing the amount of mechanical ventilation and natural ventilation, rigorous and regular cleaning and disinfection practices, frequent hand hygiene, and appropriate PPE can effectively reduce SARS-CoV-2 contamination.

### Conclusion

The present study showed a high success rate in detecting the presence of SARS-CoV-2 RNA in a makeshift COVID-19 hospital setting. The cross-contamination and persistence of SARS-CoV-2 RNA across indoor surroundings were common. It is urgent to explore the intricate relationship between environmental contamination and transmission of SARS-CoV-2 through novel approaches and perspectives. Studying the details of environmental contamination status by SARS-CoV-2 RNA in a makeshift COVID-19 hospital could help us to better understand the routes of contamination of SARS-CoV-2, and to identify the major modes of transmission, and eventually be able to mitigate the pandemic caused by COVID-19. The present findings can be used to guide the makeshift hospital infection control strategies and mitigation measures by identifying high-risk contamination locations.
